# Over-the-counter analgesics use is associated with pain and psychological distress among adolescents: a mixed effects approach in cross-sectional survey data from Norway

**DOI:** 10.1186/s12889-021-12054-3

**Published:** 2021-11-06

**Authors:** Rune Jonassen, Eva Hilland, Catherine J. Harmer, Dawit S. Abebe, Anne Kristine Bergem, Siv Skarstein

**Affiliations:** 1 Faculty of Health Sciences, Oslo Metropolitan University, Pilestredet 32, 0166 Oslo, Norway; 2 NORMENT, Department of Medicine, University of Oslo, Oslo, Norway; 3 Psychopharmacology and Emotion Research Laboratory, Department of Psychiatry, University of Oxford, Oxford, UK; 4 Oxford Health NHS Foundation Trust, Warneford Hospital, Oxford, OX3 7JX UK

**Keywords:** Over-the-counter analgesics, Psychological distress, Pain, Sex differences

## Abstract

**Background:**

Over-the-counter analgesics (OTCA) such as Paracetamol and Ibuprofen are frequently used by adolescents, and the route of administration and access at home allows unsupervised use. Psychological distress and pain occur simultaneously and are more common among females than among males. There is a dynamic interplay between on-label pain indications and psychological distress, and frequent OTCA use or misuse can exacerbate symptoms. No studies have to date provided an overview of frequent OTCA use in a larger population-based study. The current study used survey data to explore associations between and the relative predictive value of on-label pain indication and measures of psychological distress, together with sex differences for weekly OTCA use.

**Methods:**

This study included 349,528 adolescents aged 13–19. The data was collected annually between January 2014 and December 2018 as part of the Norwegian Young Data survey. Performance analysis was conducted to explore the relative roles and associations between on-label pain indication and psychological distress in weekly OTCA use. A mixed-effects logistic regression model was used to explore the unique contributions from four domains of on-label pain indication and psychological distress as measured by a combined measure of anxiety and depression (HSCL-10) and peer-bullying involvement as victims or bullies.

**Results:**

Thirty percent of females and 13 % of males use OTCA weekly. Headache is the strongest on-label pain predictor of weekly OTCA use, followed by abdominal pain. Depression and anxiety are the strongest psychological predictor of weekly OTCA use, and higher symptom levels and being female increase the strength of this association. Anxiety and depression also predict weekly OTCA use after controlling for physiological pain.

**Conclusions:**

Sex, pain and anxiety and depression are inter-correlated and strong predictors of frequent OTCA use. Frequent OTCA use in the context of psychological distress may be a form of self-medication that can exacerbate symptoms and decrease psychosocial function. Longitudinal studies that explore causal trajectories between frequent on-label OTCA use and psychological distress are required. OTCA use among adolescents, and particularly among females, with anxiety and depression should be administered with caution and closely monitored.

## Background

Paracetamol (acetaminophen) and Ibuprofen are available as over-the-counter analgesics (OTCA) and are among the most widely used pharmacological agents of our time. Paracetamol, also known as acetaminophen, is a medication used to treat pain and fever**. Acetaminophen** is the major metabolite of acetanilide and phenacetin responsible for the analgesic effects [1–3]. Ibuprofen is a **nonsteroidal anti-inflammatory drug (NSAID)** used to reduce fever and to treat pain or inflammation. Ibuprofen works by blocking an enzyme that makes prostaglandin (a hormone-like substance that participates in a variety of body functions), which results in lower levels of prostaglandins in the body [3, 4]. Both OTCAs are on the World Health Organization’s list of essential medicines [5]. Efficacy is extensively documented and the safety profiles of several specific indications are well described in the literature [6, 7]. There is no evidence from randomized controlled trials to support or refute the use of Paracetamol [8] or Ibuprofen [9] to treat chronic forms of pain in children and adolescents, and no conclusions can be made about either efficacy or harm. A recent evaluation found multiple inconsistencies, heterogeneity and very narrow topics in the existing systematic reviews on Paracetamol and Ibuprofen use among children and adolescents up to the age of eighteen [10] and safety profile evaluations therefore require a broader scope. OTCA abuse is broadly defined as the systematic overuse of non-prescription medicine, and it is a serious global health challenge [11, 12].

Self-administration of OTCA starts early in life and most adolescents have access at home [13]. Few studies have provided descriptions of frequent OTCA use based on self-reports, and the subjective experiences that lead up to OTCA use and misuse remain largely unexplored. The proportion of daily and weekly users of OTCA among adolescents is rapidly increasing [14–17]. Approximately 25% use OTCA at least weekly in adolescence and these high consumers report lower self-esteem, reduced sleep, lower educational ambition, binge drinking, higher caffeine consumption, and part-time employment when they are compared to non-weekly users [18].

OTCA use has been linked to several forms of psychological and psychosocial stressors. The association between OTCA use and perceived stress has been reported [19]. Victims of peer-bullying are associated with OTCA use, even when controlled for the higher prevalence of pain among victims [20]. A single dose of Paracetamol reduced affective reactivity to other people’s positive experiences in adolescents and suggests that the mechanisms of action may have a negative impact on prosocial behavior [21]. Daily use of Paracetamol reduces behavioral and neural responses associated with the pain of social rejection [22]. Several studies have shown that OTCA may influence how people experience distress, process cognitive discrepancies and evaluate stimuli in their environment [23]. Therefore, high OTCA consumption is likely to be linked to several factors outside the somatic sphere.

Pain is complex and involves both biological, psychological and psychosocial mechanisms. Psychological distress crosses traditional diagnostic boundaries by affecting both mental and physical health [24]. Depression, anxiety and pain are on the rise among adolescents [25, 26], and there is also increasing use of analgesics [27]. A few recent studies have investigated psychiatric symptoms, pain and analgesics in youth. Headaches and abdominal pain were reported more often by adolescents with high levels of psychiatric symptoms [28]. Females with depressive symptoms tend to use more analgesic drugs compared with those who only experience pain, while those who experience pain combined with depressive symptoms take pain medication twice as often [27]. A recent survey-based study reported frequent pain and depressive symptoms among school-aged adolescents [29]. Pain and depressive symptoms were more pronounced in females than in males, and pain and depressive symptoms were related to each other. Another recent study investigated depressive symptoms, pain and the use of analgesics, and found that depressive symptoms are significantly associated with analgesics use among adolescents even after controlling for pain [30].

Depression shows high comorbidity rates with anxiety via multiple pathways [31, 32], and both conditions are associated with pain [33]. Depression and anxiety are major risk factors of suicide in adolescents and in the general population [34–37]. Adolescent victims of bullying have an elevated risk of suicidal ideation and attempts, and this association is mediated by depression, sex and lack of social support [38]. Paracetamol is the most frequently ingested compound in intentional overdosing and causes liver failure [39, 40]. Adolescent females are more likely to report deliberate self-poisoning with Paracetamol [41].

Inflammation is involved in depression and anti-inflammatories like Ibuprofen may be taken as a way of self-medication. Depression have been linked to alterations in inflammatory markers in adults [42]. Antidepressants have been shown to decrease inflammation and higher levels of inflammatory markers is associated with lower treatment responses [43], thought there is no evidence to support OTCA usefulness as treatment against depression in adolescence.

Both headaches and abdominal pain often co-occur with hormonal fluctuation in the menstrual cycle as well as mood changes. A recent meta-analysis concluded that Ibuprofen was the most effective OTCA for dysmenorrhea [44]. Females may therefore differ from males in trajectories that lead up to frequent OTCA use.

The studies described above provide evidence that the dynamic interplay between on-label pain indication, psychological distress and sex differences predict frequent OTCA use among adolescents. The causal relationships between psychosocial distress, pain and frequent OTCA use is complex, and is probably also hampered by on-label descriptions that confound self-reports. There is a lack of evidence showing that the relative role of on-label pain indications and psychological distress in frequent OTCA use will help in clinical monitoring, including in preventing suicides and medication-induced pain, which represents a major knowledge gap in the literature. No studies to date have described either on-label or off-label frequent OTCA use in large population-based studies. Therefore, the associations between the most frequently used pharmacological agents of our time and the relative impact of factors linked to use and misuse remain largely unexplored. The objectives of the current study were therefore to describe the relative role of on-label pain indication, psychological distress and sex differences in weekly OTCA use. The predictive value of on-label pain domains was explored, and a combined measure of anxiety and depression was compared to peer-bullying involvement to highlight how these domains of psychological distress are related to weekly OTCA use.

## Methods

### Participants

The Young Data Survey (Ungdata) is a cross-sectional and national data collection scheme, designed to conduct surveys of adolescents in Norway at the municipality level. A sample of 349,528 adolescents was included and the data was collected annually over five years between January 2014 and December 2018 in high schools among students aged 13–19. Participants filled in an online questionnaire during school hours. Data was collected across seven geographical regions (South-East, Oslo (the capital), South-West, West, North-West, Middle, and North) and includes both rural, sub-urban and urban regions of Norway. The interval between assessments within the same area is three years, and there is no response option in the survey that inform the study about earlier participation.

#### Methods and measurements

##### Over-the-counter analgesics

The frequency of using OTCA (Paracetamol, Ibuprofen or similar) in the last month was measured using the response options 1- never, 2- less than once a week, 3- at least weekly, 4- more times during a week and 5 – daily. The response options 3–5 indicate at least weekly OTCA use. Paracetamol and Ibuprofen are the most sold OTCAs and rank second and third after nicotine medication sold in Norway. There is an age and quantity restriction (18 years and one package) for OTCA sold in stores, newsstands and gas stations. There are no age restrictions on pharmacies selling OTCAs in Norway, but they are obliged to provide guidance on use, side effects and misuse. Consumers may only purchase one package (20 tablets a 500 mg Paracetamol or 200 mg Ibuprofen) at a time.

##### On-label pain indication

Four on-label indications for OTCA were included in the survey and are used in the current study; 1) Headache, 2) Abdominal pain, 3) Muscle and joint pain, and 4) Neck and shoulder pain. The survey asks adolescents to rate how often they have experienced these symptoms during the last month with the response options 1- not at all, 2- sometimes, 3- many times, and 4- daily.

##### Psychological distress

The Hopkins Symptom Checklist (HSCL-10) was used as a measure of psychological distress related to anxiety and depression. HSCL-10 is a short version of HSCL-25, and HSCL-10 performs almost as well as the full version for adolescents aged ≥15 years. A very high correlation (0.97) between the HSCL-25 and the HSCL-10 was found, with a sensitivity of 89% and a specificity of 98% for HSCL-10 using the HSCL-25 (cut-off 1.75) criterion [45]. AL Kleppang and C Hagquist [46] have provided a detailed description of the psychometric properties of HSCL-10 in relation to Norwegian adolescents. The questionnaire consists of four anxiety items; 1) Suddenly scared for no reason 2) Feeling fearful 3) Faintness, dizziness, or weakness, and 4) Feeling tense or keyed up, and six depression items; 5) Blaming yourself for things, 6) Difficulty in falling asleep or staying asleep, 7) Feeling blue, 8) Feeling of worthlessness, 9) Feeling everything is an effort, and 10) Feeling hopeless about the future. Adolescents are asked to rate symptoms during the preceding week with the response options; 1- not at all, 2- a little bit, 3- quite a bit and 4- extremely. Incidents related to bullying, either as the bully and/or the victim, were assessed by the two items; 1) Are you involved in teasing, threatening or excluding other young people at school or during leisure time? and 2) Are you yourself subjected to harassment, threats or exclusion by other young people at school or during leisure time? Values were set based on a 6-point scale with the response options; 1-many times during a week, 2- at least once a week, 3- at least once in the last two weeks, 4- at least once a month, 5- seldom and 6- never. The response options 1–4 defined victims and bullies respectively.

#### Data preparation and statistical analysis

All data were analyzed and visualized in RStudio-version 1.3.959. HSCL-10 was treated as a three-level factor where the mean score was calculated for participants who had completed HSCL-10 data for at least eight of the ten items. Only participants with OTCA data were included in the analysis. A total of 297,480 (85%) participants met this criterion. The outcome variable OTCA was calculated as dichotomous where the responses 3 through 5 were defined as weekly OTCA use. Psychological distress was treated as a factor (average of 10 variables and three levels) where scores between 1 and 2 = minimal, − 2 and 3 = moderate, and 3 and 4 = severe.

On-label indications were treated as factors (four variables and four levels). Geographical region (7) was treated as a random factor together with year of study (5 years). Sex was coded as 0 = males and 1 = females. The magnitude of predictor variables for the data point relative to the maximum magnitude of the predictor variables across all data points was visualized in a radar chart. We used the function *ggRadar* which rescales all variables to have a minimum of 0 and a maximum of 1.

Performance analysis was conducted by using the *chart. Correlation* function that provides a visualization of distribution patterns and correlation statistics *(method = spearman)* between variables. The performance analysis provides information about which factor levels that drive the correlations, by combining a traditional correlation matrix with the distribution of variables with a fitted line.

The *glmer* function was used to fit mixed-effects logistic regression models. Before fitting the models, the absolute and relative frequency of values within each factor was calculated together with proportions of missing values. Diagnostics for the random factors (geographical region and year of assessment) were run and plotted as standard normal quantiles against random effect quantiles. **M0** used two random factors only to predict weekly OTCA use. **M1** used the random factors and the four on-label pain indications to predict weekly OTCA use and thereafter added sex as an interaction term. **M2** used the HSCL-10, bully and victim of bullying as psychological distress predictors for weekly OTCA use and subsequently added sex as an interaction term. Model outcomes were presented as Odds Ratio and 95% CI per predictor, and model performance was evaluated based on Marginal and Conditional R^2^. Estimations of model fit and complexity used *anova* and were evaluated using the Akaike information criterion (AIC) and the Bayesian information criterion (BIC) against the random intercept-only model (M0). Marginal and Conditional R^2^ is provided for each model. **M3** used all variables in M1 and M2 to explore the predictive value of psychological distress after controlling for on-label pain indication.

## Results

### Descriptive statistics

**Fig. 1:**
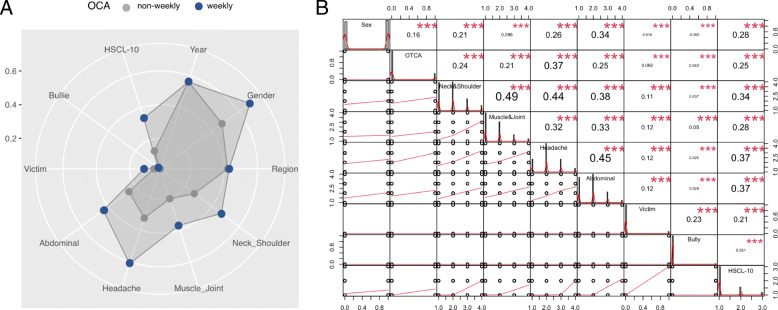
**a** Shows radar chart with relative impact of variables after value standardization. **b** Shows performance analysis with correlation matrix (above right), factor value frequencies (middle diagonal) and linear fit (bottom left). ***= *p* < .001

A total of 61,485 (17.6%) adolescents used OTCA at least weekly and 288,043 (82.4%) were non-weekly users. Females made up 171,363 (50.8%), and males 166,076 (49.2%) of the sample. The proportion of weekly OTCA use among females was 30.3 and 13.2% among males. The relative differences linked to weekly OCA user versus non-weekly users were observable for HSCL-10, and for the four on-label pain indications. There are relatively small differences related to peer-bullying involvement. No differences are observed for assessment year or geographical region (Fig. 1a).

The strongest correlations were found between on-label pain indications for OTCA, between weekly OTCA use and headache, and between HSCL-10 and on-label indications. The association between HSCL-10 and OTCA use is similar to associations between OTCA and the other three on-label indications (neck and shoulder pain, muscle and joint pain, and abdominal pain). The strongest associations with sex are found for abdominal pain, followed by HSCL-10 and headache. The pattern of the association between pain indication and HSCL-10 appears to be non-linear and indicates that this association is manifested when individuals experience pain more often. Among the psychological distress variables, HSCL-10 shows higher correlation with other variables than being involved in peer-bullying as bully or victim (Fig. 1b).

#### Relative OTCA group differences and variable performance analysis

##### Results of mixed-effects logistic regression

**Fig. 2:**
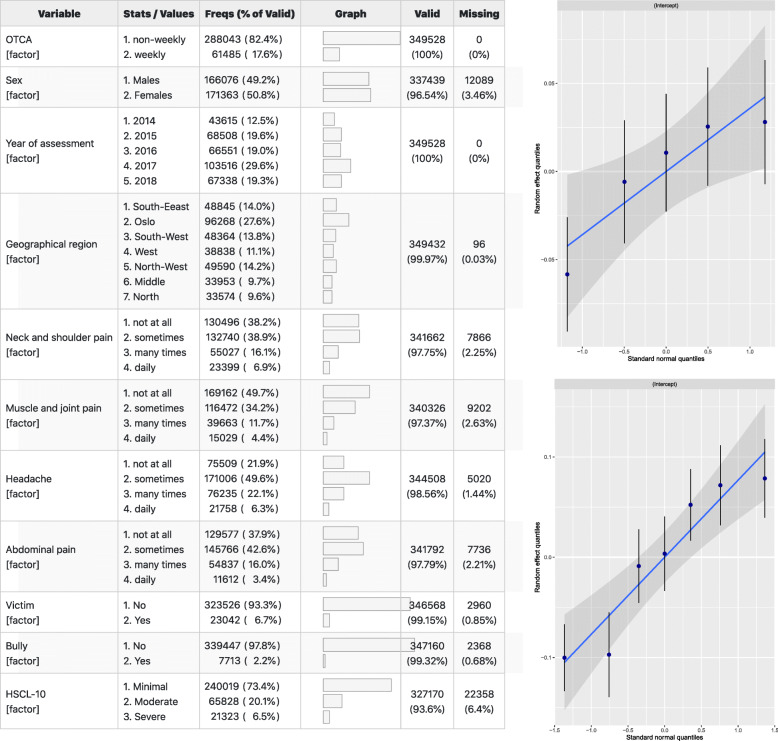
Shows the frequency and proportion of values per factor with number and percentage missing (left). The diagnostic plots (right) show diagnostics for variables used as random factors

Frequency variables used for model estimations showed that 6.5% report more severe degrees of anxiety and depression. We also found that sometimes experiencing headaches during the preceding week was more common than not experiencing headaches at all. A similar, but less pronounced pattern is observed for abdominal pain. Oslo (region 2.) contributes more to the random factor variance (Q^2^) than other geographical regions (Fig. 2).

#### Descriptive statistics and diagnostic plots

##### M1. Weekly OTCA use predicted by on-label pain indications with random effect

The model shows that headache is the strongest predictor of weekly OTCA use and that those who very often experience headaches use OTCA 17.7 times more often than non-weekly OTCA users. The model explains about 27% of the total variance. Being female is associated with more OTCA use related to all on-label predictors, except when they experience mild forms of muscle and joint pain. Headache and abdominal pain are the predictors that increase the most by being female. The model that included sex interaction explained about 34% of the total variance (Table 1).

**Table 1:** shows OR and 95% CI per factor level for on-label pain indication. ICC = intraclass correlation coefficient. σ^2^ = random intercept variance

	*Odds Ratio*	*CI*	*p*
***OTCA weekly***			
(Intercept)	0.04	0.03–0.04	**< 0.001**
Neck and shoulder pain 2	1.11	1.08–1.14	**< 0.001**
Neck and shoulder pain 3	1.26	1.22–1.31	**< 0.001**
Neck and shoulder pain 4	1.27	1.21–1.33	**< 0.001**
Muscle and joint pain 2	1.20	1.17–1.23	**< 0.001**
Muscle and joint pain 3	1.44	1.39–1.49	**< 0.001**
Muscle and joint pain 4	1.86	1.77–1.96	**< 0.001**
Headache 2	1.90	1.82–1.98	**< 0.001**
Headache 3	7.17	6.86–7.49	**< 0.001**
Headache 4	17.78	16.87–18.73	**< 0.001**
Abdominal pain 2	1.31	1.28–1.35	**< 0.001**
Abdominal pain 3	1.88	1.82–1.95	**< 0.001**
Abdominal pain 4	2.24	2.12–2.36	**< 0.001**
**Random effects**			
σ^2^	3.29		
ICC	0.01		
N _Region_	7		
N _Year_	5		
Observations	297,480		
Marginal R^2^ / Conditional R^2^	0.267 / 0.271		
OTCA weekly * Sex			
(Intercept)	0.01	0.01–0.01	**< 0.001**
Neck and shoulder pain 2	1.17	1.13–1.21	**< 0.001**
Neck and shoulder pain 3	1.50	1.44–1.57	**< 0.001**
Neck and shoulder pain 4	1.58	1.50–1.67	**< 0.001**
Muscle and joint pain 1	1.02	0.99–1.05	0.197
Muscle and joint pain 2	1.12	1.08–1.17	**< 0.001**
Muscle and joint pain 3	1.30	1.23–1.38	**< 0.001**
Headache 2	2.30	2.16–2.45	**< 0.001**
Headache 3	8.55	8.02–9.12	**< 0.001**
Headache 4	21.91	20.44–23.49	**< 0.001**
Abdominal pain 2	2.14	2.05–2.22	**< 0.001**
Abdominal pain 3	3.83	3.68–4.00	**< 0.001**
Abdominal pain 4	3.88	3.66–4.12	**< 0.001**
**Random effects**			
σ^2^	3.29		
ICC	0.01		
N _Region_	7		
N _Year_	5		
Observations	297,480		
Marginal R^2^ / Conditional R^2^	0.342 / 0.346		

##### M2. Weekly OTCA use predicted by psychological distress with random effects

The model shows that anxiety and depression is a strong predictor of weekly OTCA use. The model explained about 10% of the total variance. This effect is stronger in females than in males. Including the sex interaction in the model increased the predictive value to about 15%. Females who have high degrees of anxiety and depression use OTCA about 9.5 times more than the non-weekly users who are males. The proportion of OTCA use associated with anxiety and depression doubles per severity level (minimal, moderate and severe) (Table 2.).

**Table 2:** shows OR and 95% CI per factor level for psychological distress. ICC = intraclass correlation coefficient. σ^2^ = random intercept variance

	*Odds Ratio*	*CI*	*p*
**OTCA weekly**			
(Intercept)	0.14	0.12–0.15	**< 0.001**
Victim of bullying	1.26	1.21–1.30	**< 0.001**
Bully	1.42	1.34–1.51	**< 0.001**
HSCL-10 2	2.95	2.89–3.02	**< 0.001**
HSCL-10 3	6.00	5.81–6.19	**< 0.001**
**Random Effects**			
σ^2^	3.29		
ICC	0.00		
N _Region_	7		
N _Year_	5		
Observations	297,480		
Marginal R^2^ / Conditional R^2^	0.098 / 0.101		
OTCA weekly * Sex			
(Intercept)	0.07	0.06–0.08	**< 0.001**
Victim of bullying	1.12	1.08–1.17	**< 0.001**
Bully	0.64	0.59–0.70	**< 0.001**
HSCL-10 2	4.21	4.11–4.32	**< 0.001**
HSCL-10 3	9.44	9.12–9.78	**< 0.001**
**Random Effects**			
σ^2^	3.29		
ICC	0.00		
N _Region_	7		
N _Year_	5		
Observations	297,480		
Marginal R^2^ / Conditional R^2^	0.145 / 0.149		

### Estimations of model fit and complexity

Predicting OTCA*Sex by on-label indication (M1) performed better that the random effects only model [M0| M1; (AIC = 274,684; BIC = 274,715) | (AIC = 168,854; BIC = 168,928, *X*^*2*^_=_ 10,583, *P* < .001)]. Predicting OCA*Gender by psychological distress was superior to the random effect model [M0; (AIC = 194,762; BIC = 194,825, *X*^*2*^_=_ 79,928, *P* < .001)]. The on-label model performed relatively better than the psychological distress model [M2| M3; (AIC = 168,854; BIC = 168,928) | (AIC = 194,762; BIC = 194,825, *X*^*2*^_=_ 25,909, P < .001)].

#### M3. Weekly OTCA use predicted by on-label pain indications and psychological distress with random effects

The model shows the culmination of all the factors from M1 and M2. The odds ratio for moderate degrees of symptoms was (OR = 1.42, 95% = 1.38, 1.45, *p* < .001) and (OR = 1.69, 95% CI = 1.62, 175, *p* < .001) for severe degrees of symptoms across sexes, and was (OR = 1.87, 95% CI = 1.81, 1.92, p < .001) for moderate degrees of symptoms and (OR = 2.45, 95% CI = 2.35,255, p < .001) for severe degrees of symptoms in females (R^2^ = .356) (Table 3).

**Table 3:** shows OR and 95% CI per factor level for on-label pain indication and psychological distress. ICC = intraclass correlation coefficient. σ^2^ = random intercept variance

	*Odds Ratio*	*CI*	*P*
**OTCA weekly**			
(Intercept)	0.04	0.03–0.04	**< 0.001**
Neck and shoulder pain 2	1.09	1.06–1.12	**< 0.001**
Neck and shoulder pain 3	1.19	1.15–1.23	**< 0.001**
Neck and shoulder pain 4	1.17	1.12–1.23	**< 0.001**
Muscle and joint pain 2	1.17	1.14–1.20	**< 0.001**
Muscle and joint pain 3	1.37	1.33–1.42	**< 0.001**
Muscle and joint pain 4	1.74	1.66–1.83	**< 0.001**
Headache 2	1.87	1.79–1.95	**< 0.001**
Headache 3	6.74	6.45–7.04	**< 0.001**
Headache 4	15.70	14.90–16.56	**< 0.001**
Abdominal pain 2	1.26	1.23–1.30	**< 0.001**
Abdominal pain 3	1.69	1.63–1.75	**< 0.001**
Abdominal pain 4	1.90	1.80–2.00	**< 0.001**
HSCL-10 2	1.42	1.38–1.45	**< 0.001**
HSCL-10 3	1.69	1.62–1.75	**< 0.001**
Victim	1.04	1.00–1.08	**0.048**
Bully	1.45	1.35–1.55	**< 0.001**
**Random effects**			
σ^2^	3.29		
ICC	0.01		
N _Region_	7		
N _Year_	5		
Observations	297,484		
Marginal R^2^ / Conditional R^2^	0.272 / 0.276		
OTCA weekly * Sex			
(Intercept)	0.01	0.01–0.01	**< 0.001**
Neck and shoulder pain 2	1.12	1.08–1.17	**< 0.001**
Neck and shoulder pain 3	1.36	1.31–1.42	**< 0.001**
Neck and shoulder pain 4	1.39	1.31–1.46	**< 0.001**
Muscle and joint pain 2	0.98	0.95–1.02	0.348
Muscle and joint pain 3	1.05	1.01–1.09	**0.017**
Muscle and joint pain 4	1.21	1.14–1.28	**< 0.001**
Headache 2	2.22	2.08–2.37	**< 0.001**
Headache 3	7.60	7.12–8.10	**< 0.001**
Headache 4	17.86	16.65–19.16	**< 0.001**
Abdominal pain 2	1.99	1.91–2.07	**< 0.001**
Abdominal pain 3	3.20	3.07–3.35	**< 0.001**
Abdominal pain 4	3.07	2.88–3.26	**< 0.001**
HSCL-10 2	1.87	1.81–1.92	**< 0.001**
HSCL_10 3	2.45	2.35–2.55	**< 0.001**
Victim	0.90	0.86–0.94	**< 0.001**
Bully	0.55	0.50–0.61	**< 0.001**
**Random effects**			
σ^2^	3.29		
ICC	0.01		
N _Region_	7		
N _Year_	5		
Observations	297,484		
Marginal R^2^ / Conditional R^2^	0.351 / 0.356		

## Discussion

In the Young Data Survey, the prevalence of weekly OTCA use was 17.6% across the whole sample, and 30% in females. Headache is by far the strongest on-label predictor of weekly OTCA use. Abdominal pain is the second-best on-label predictor of weekly OTCA use, and the role of both headache and abdominal pain is more pronounced in females than in males. Weekly OTCA use is particularly common and increases exponentially as adolescents experience headaches more often, while the much smaller increase in weekly OTCA use related to abdominal pain is also found when adolescents experience symptoms more rarely. The proportion of severe anxiety and depression in weekly OTCA users is six times higher than in non-weekly users across sexes and increases to almost ten times in female weekly users. Weekly OTCA use predicted by anxiety and depression is proportional and doubles with severity levels, and shows that individuals with moderate symptoms are also weekly OTCA users three times more often than those with minimal symptoms. Adolescents with more severe anxiety and depression also use OTCA weekly about 1.7 times more often across sexes and about 2.5 times more often in females even after controlling for pain and peer-bullying involvement.

The results of this performance analysis are in line with previous evidence showing a considerable overlap between pain and psychological distress [47, 48] and are also in accordance with the literature that shows that females generally report more pain, anxiety and depression [49–52]. Among psychological distress variables, the combined measure of anxiety and depression (HSCL-10) is more related to all other variables than peer-bullying involvement. The unique effects of being involved in peer bullying as a victim or a bully was small.

The links between anxiety and depression and pain are observed to be specific to combinations of the frequency of experienced pain and anxiety and depression severity levels. We did not observe large OTCA differences linked to geographical region or year of study. These findings have important implications for operationalization and analysis in this and further studies that aim to explore the unique impact of psychological distress, pain and OTCA use. The current study took the inter-correlations into account by modelling psychological stress predictors separately. On-label indications are generally thought to be much stronger predictors than off-label use and setting these domains up against each other may be conceptually problematic. The results of the statistical models in this study provide evidence that shows a unique link between anxiety and depression, and OTCA use, which is not found in other domains of psychological distress. Importantly, the data is cross-sectional and should not be interpreted as evidence of OTCA use as self-medication for psychological complaints in absence of pain. However, the observed presence of psychological distress in the context of weekly OTCA use has important clinical implications. Adolescence is a period of biologically-driven developmental transition of puberty, which has secondary effects on social, emotional and sexual development. The findings from this study show that anxiety and depression play a key role in frequent OTCA use among young people. Notably, continuous use of OTCA as a means to combat pain and avoid stress can prevent adolescents from learning healthier coping strategies, as such behavioral patterns are likely to progress into adulthood [30, 53].. Given the emerging evidence that shows negative OTCA effects in psychological, social functioning and suicide risk, frequent OTCA use, misuse and route of administration should be monitored closely by parents, health services and policy makers. The proximal risk of suicide is greatest when depression and anxiety co-occur [54] and the current study shows that a combined measure of anxiety and depression is sensitive in predicting frequent OTCA use.

Medication Overuse Headache (MOH) is a subtype of chronic daily headache caused by overuse of one or more analgesics. MOH prevalence is estimated in about 1–2% of the general population [55], and is more prevalent in women than in men [56–58]. Recent studies show that MOH is common in pediatric populations [59–62]. MOH may be among the reasons why headache dominates among on-label pain indications in the current study. Frequent OTCA use for headaches and other forms of pain, can cause chronic headaches, and frequent use of Ibuprofen, anxiety, and depression, and being female are among the factors that increase the risk of OTCA-induced chronic headaches [63, 64]. Reports of the overall prevalence of self-reported chronic pain in adolescence is high. A recent study showed that about 45% of those aged between 11 and 15 experienced chronic weekly pain during a six-month period. The prevalence of weekly headaches was 11.3% and was generally more common in females across most countries [65]. Adolescents’ chronic pain management is therefore a major health challenge and the current study suggests that frequent use of OTCA may be a preferred coping strategy. Coping strategies are learned and often passed on in new situations, and whether or not they produce successful outcomes is not in itself decisive [66]. If a young person has learned that using OTCA is a good way to deal with pain, analgesic medication may become her preferred solution to resolve many painful situations, including psychological distress.

Hereunder, a recent review concludes that parents are the most important source of information regarding the use of OTCA in adolescence and are also the main supplier of the medicine [67].

The current study has several imitations that should be mentioned. A wide definition of OTCA is employed as no specific questions in the survey ask for the type of OTCA. The definition of OTCA does not take prescriptions from health services into account. Analgesics sold over the counter can also be introduced as treatment by primary and specialist healthcare services, which are also allowed to prescribe higher doses. The survey was conducted within school hours and chronic forms of pain may affect the degree of school attendance, and therefore also influence compliance in the current study. The results may have relevance to other forms of self-medication in different cultural settings, like the use of cannabinoids, in countries where these drugs are legal and widely available. Longitudinal studies will help explain causal trajectories that underlie associations between psychological distress and frequent OTCA use between sexes and should also include an assessment of the female menstrual cycle. The study was conducted in a large sample, and it addressed and revealed unobserved sex differences, and used performance analysis prior to conducting mixed-effects logistic regression modelling which is a strength.

## Conclusions

Headache is the dominant on-label indication related to weekly OTCA use in adolescence followed by abdominal pain. Females more often use OTCA at least weekly when they experience headache and abdominal pain. Anxiety and depression are associated with pain, and adolescents with a more severe degree of symptoms more often use OTCA at least weekly. This relative proportion is also larger in females with more severe degrees of symptoms who use OTCA at least weekly ten times more often than those with minimal symptoms. The current study provides evidence that requires health professionals to be careful when assessing OTCA use in adolescents with anxiety and depression.

## Data Availability

The datasets analyzed during the current study are not publicly available due to lack of consent to sharing individual data. Meta data is available from the corresponding Young Data Survey on reasonable request. Data and material are stored at Oslo Metropolitan University P.O. Box 4, St. Olavs plass. N-0130 OSLO, Norway.

## Abbreviations

OTCA: Over-the-counter analgesics
HSCL-10: The Hopkins Symptom Checklist − 10
NSAID: Nonsteroidal anti-inflammatory drug
AIK: Akaike information criterion
BIC: Bayesian information criterion
MOH: Medication Overuse Headache
